# A new gall crab species (Brachyura, Cryptochiridae) associated with the free-living coral *Trachyphyllia geoffroyi* (Scleractinia, Merulinidae)

**DOI:** 10.3897/zookeys.500.9244

**Published:** 2015-04-27

**Authors:** Sancia E.T. van der Meij

**Affiliations:** 1 Department of Marine Zoology, Naturalis Biodiversity Center, Darwinweg 2, 2333 CR Leiden, The Netherlands

**Keywords:** Cospeciation, host specificity, Indonesia, Malaysia, Thoracotremata

## Abstract

A new species of gall crab is described from the free-living stony coral *Trachyphyllia geoffroyi*. Specimens were collected during field work in Lembeh Strait (Indonesia) and off Kudat (Malaysian Borneo). This new species, here named *Lithoscaptus semperi* **sp. n.**, is the ninth species assigned to the genus. It can be separated from its congeners by not having the internal orbital angle extending beyond the external orbital angle, and by the stout female P2 merus with prominent distomesial projection. In addition, the carapace surface appears smooth, despite having small tubercles on the anterior half, and is without noticeable spines, other than those on the frontal margin. The distinctive carapace pattern in life is a diagnostic character in male specimens.

## Introduction

During field work in Indonesia and Malaysia an undescribed gall crab species was encountered living in dwellings in free-living *Trachyphyllia geoffroyi* (Audouin, 1826) corals. This scleractinian species is usually found on soft substrate of reef bases near coral reefs, where it can occur in large numbers (Fisk 1983, Best and Hoeksema 1987). The polyps of *Trachyphyllia geoffroyi* are fleshy and a large mantle can extend beyond the perimeter of the skeleton.

*Trachyphyllia geoffroyi* was classified in its own family, Trachyphylliidae Verrill, 1901, but this taxon was recently synonymised with Merulinidae Verrill, 1865 (Huang et al. 2014). The sister genera of *Trachyphyllia* Milne Edwards & Haime, 1849 are *Coelastrea* Verrill, 1866 and *Dipsastraea* de Blainville, 1830, which include coral species that formerly belonged to *Goniastrea* Milne Edwards & Haime, 1848 and *Favia* Milne Edwards, 1857. Corals belonging to these genera are host to cryptochirids of the genus *Lithoscaptus* A. Milne-Edwards, 1862 (Fize and Serène 1957, Kropp 1990).

Semper (1881) mentioned gall crabs associated with Indo-Pacific and Atlantic “*Trachyphyllia*”, but no formally described gall crab has been recorded living in association with *Trachyphyllia geoffroyi*. This new gall crab species, here named *Lithoscaptus semperi* sp. n., is the ninth assigned to the genus.

## Methods

Gall crabs were collected in Indonesia (Lembeh Strait, N Sulawesi – February 2012) and Malaysia (off Kudat, N Borneo – September 2012). Corals were searched for gall crabs, taken to the field laboratory and subsequently split with hammer and chisel. The crabs were preserved in 80% ethanol, after being photographed with a digital SLR camera equipped with a macro lens to register colour patterns. All crab specimens are deposited in the Crustacea collection of Naturalis Biodiversity Center in Leiden, the Netherlands (formerly Rijksmuseum van Natuurlijke Historie, collection coded as RMNH.Crus.D).

Drawings were made with a stereomicroscope with camera lucida. Carapace lengths and widths were measured to the nearest 0.1 mm using an eyepiece micrometre, with the crabs positioned on a level surface. Abbreviations used: CL, carapace length; CW, carapace width (at widest point); MXP3, third maxilliped; ovig., ovigerous; P, pereiopod; G, male gonopod. Carapace measurements are given as CL × CW, in mm.

## Taxonomy

### Cryptochiridae Paul′son, 1875
*Lithoscaptus* A. Milne-Edwards, 1862

#### Lithoscaptus semperi sp. n.

Taxon classificationAnimaliaDecapodaCryptochiridae

http://zoobank.org/65F0D837-961A-42B7-8F9E-2C806DD54238

Figs 1 , 2 , 3

##### Type locality.

Tigabu Isl. (06°53'51"N, 117°27'36"E), Kudat, Sabah (N Borneo), Malaysia.

##### Coral host holotype.

*Trachyphyllia geoffroyi* (Audouin, 1826).

##### DNA barcoding.

A COI sequence (partially, Folmer et al. 1994) of paratype RMNH.Crus.D.54331 has been deposited in GenBank under accession number KP688583.

##### Type material.

**Holotype.** RMNH.Crus.D.56962, ovig. female, 6.4 × 4.6. **Allotype** (with holotype), male, 3.6 × 2.5. Collected by the author from 13 m depth on 8 September 2012. **Paratype.** RMNH.Crus.D.54331, Lubani Rock, Kudat, Sabah (N Borneo), Malaysia (06°53'45.0"N, 117°23'15.8"E), 10–15 m, 07.ix.2012, 1 ovig. female, 6.2 × 4.7, leg. SET van der Meij.

##### Material examined.

**Indonesia:** RMNH.Crus.D.56957, Aer Perang, Lembeh Strait (01°28'25"N, 125°14'02"E), ca. 10 m, 02.ii.2012, 1 female, leg. BT Reijnen; RMNH.Crus.D.56958, Tanjung Labuhankompeni, Lembeh Strait (01°25'55"N, 125°11'10"E), 28 m, 04.ii.2012, 1 female, leg. BW Hoeksema; RMNH.Crus.D.56959, Kelapadua, Lembeh Strait (01°26'19"N, 125°12'49"E), 20 m, 09.ii.2012, 2 juvenile males, leg. BW Hoeksema; RMNH.Crus.D.54250, Tanjung Nanas I, Lembeh Strait (01°27'39"N, 125°13'35"E), 25–30 m, 17.ii.201, 1 ovig. female, 1 female, leg. BW Hoeksema; **Malaysia:** RMNH.Crus.D.54259, Lubani Rock, Kudat (06°53'45"N, 117°23'15"E), 10–15 m, 07.ix.2012, 1 ovig. female (slightly damaged), leg. BW Hoeksema; RMNH.Crus.D.54280, Lubani Rock, Kudat (06°53'45"N, 117°23'15"E), 10–15 m, 07.ix.2012, 1 ovig. female, 1 male, leg. BW Hoeksema; RMNH.Crus.D.56960, Lubani Rock, Kudat (06°53'45"N, 117°23'15"E), 10–15 m, 07.ix.2012, 1 male, leg. SET van der Meij; RMNH.Crus.D.56961, Lubani Rock, Kudat (06°53'45"N, 117°23'15"E), 10–15 m, 07.ix.2012, 1 ovig. female, leg. SET van der Meij; RMNH.Crus.D.54312, Tigabu Is., Kudat (06°53'51"N, 117°27'36"E), 9 m, 08.ix.2012, 1 ovig. female (damaged), 1 male, leg. SET van der Meij; RMNH.Crus.D.56963, Fairway Shoal, Kudat (07°07'06"N, 117°30'42"E), 12 m, 10.ix.2012, 1 male, leg. BT Reijnen; RMNH.Crus.D.56964, Belaruan, Kudat (07°01'50"N, 117°00'41"E), ca. 15m, 20.ix.2012, 1 male, leg. BW Hoeksema; RMNH.Crus.D.54258, Tajau, Kudat (06°59'36"N, 116°50'27"E), 21 m, 25.ix.2012, 1 female, 1 male, leg. BW Hoeksema. All material was collected from the scleractinian coral *Trachyphyllia geoffroyi*.

##### Description of female holotype.

**Figure 1.**
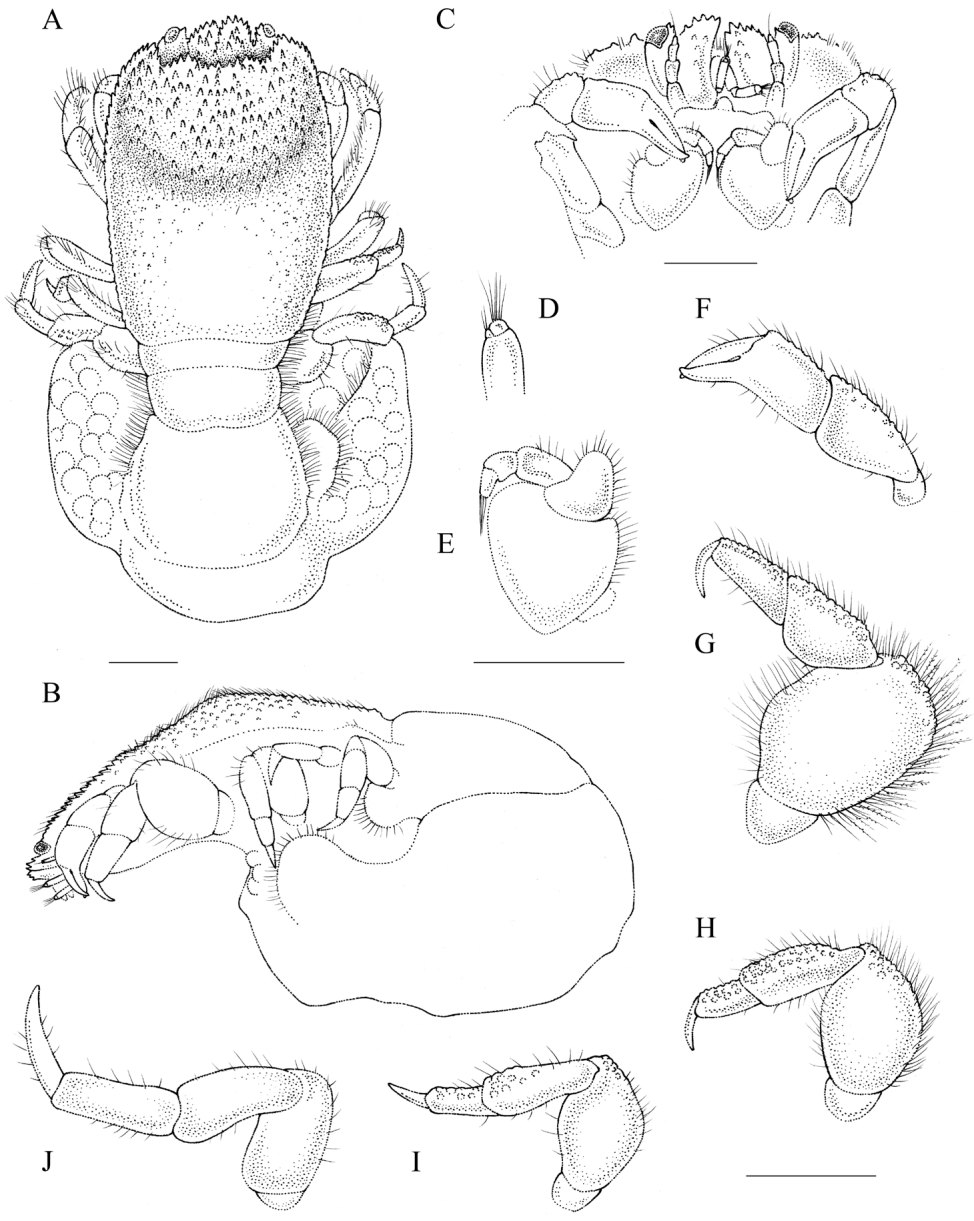
Ovigerous female holotype (6.4 × 4.6) of *Lithoscaptus semperi* sp. n. (RMNH.Crus.D.56962) **A** habitus, dorsal view **B** carapace, lateral view **C** anterior margin of carapace, ventral view **D** close-up of antennule **E** MXP3 **F** left P1 (cheliped) **G** left P2 **H** left P3 **I** left P4 **J** left P5. Scale bars 1 mm; A–B, D–E, F–J share scale bars.

Carapace (Fig. 1A) rectangular, longer than broad, CL 1.4 times longer than CW; widest near midlength, dorsal surface in lateral view strongly convex in both directions, deflected anteriorly (Fig. 1B); anterior half of carapace with small, sharp tubercles, posterior half smooth with few, rounded granules, cardiointestinal region slightly inflated. Frontal margin armed with small anteriorly directed spines. Frontal margin on ventral side features few, small tubercles. Pterygostomial region fused to carapace.

Eyestalk exposed dorsally, slightly granular, small spines on mesial margin. Cornea anterolateral. Lateral margin of stalk at same level as anterolateral angle; distal margin with small spines (Fig. 1A, C). Distal segment of antennules with protruding article, visible from ventral side (Fig. 1C, D).

Antennular peduncle dorsal surface with small, sharp tubercles, slightly inflated distomesially; apex extending beyond tip of eyestalk; spines on mesial margin larger than those on distal margin. Ventral surface smooth, slightly tapering anteriorly in ventral view (Fig. 1C).

MXP3 (Fig. 1E) exopod rectangular; ischium subtriangular, smooth, mesial and distal margins straight, anteromesial lobe with few simple setae; merus with distolateral projection, simple setae; distal portion of carpus with short, simple setae, dactylus with bundle of setae.

P1 (chelipeds, Fig. 1F) slender; carpus length twice height, scattered small tubercles on dorsal surface, simple setae; propodus length twice height, somewhat granulated, few, scattered setae, fingers slender, mesial surface of fingers smooth, cutting edge entire, tips of fingers crossing.

P2 (Fig. 1G) longer, coarser than P1; ischium without setae; merus stout, plump, smooth with few, small rounded tubercles on distal half of dorsal surface, simple setae on lateral surface, numerous plumose setae on dorsal surface; joint between merus, carpus not extending more than at right angle; carpus smooth with small rounded tubercles on dorsal surface, simple setae on dorsal surface; propodus slightly shorter than carpus, surface smooth with small rounded tubercles on dorsal surface, simple setae on lateral and dorsal surface; dactylus half-length of propodus, smooth, sharp, curved ventrally.

P3 (Fig. 1H) ischium without setae; merus length 1.5 times height, rounded, few rounded tubercles on distal half of dorsal surface, simple setae along dorsal, lateral surface; joint between merus, carpus not extending more than at right angle; carpus length 2.5 times height, rounded tubercles on dorsal surface, simple setae on lateral and dorsal surface; propodus length twice height, rounded tubercles on dorsal surface, scattered simple setae; dactylus similar length as propodus, smooth, sharp, slightly curved ventrally.

P4 (Fig. 1I) similar to P3, less coarse; ischium without setae; merus length 1.5 times height, small rounded tubercles close to joint with carpus, carpus length 2.5 times height, rounded tubercles on distal half of dorsal surface, scattered simple setae; propodus half-length carpus, rounded tubercles on distal half of dorsal surface, few scattered simple setae; dactylus similar length as propodus, smooth, sharp, straight.

P5 (Fig. 1J) ischium without setae; merus, carpus, propodus, dactylus all of equal length, all with short simple setae; carpus, propodus slender compared to merus; dactylus smooth, sharp, slightly curved ventrally.

P3, P4 decreasing in size from P2.

Abdomen enlarged, lateral margins fringed with setae (Fig. 1A, B).

Gonopore (vulva); reniform, size half the height of sternite 6.

##### Description of male allotype.

**Figure 2.**
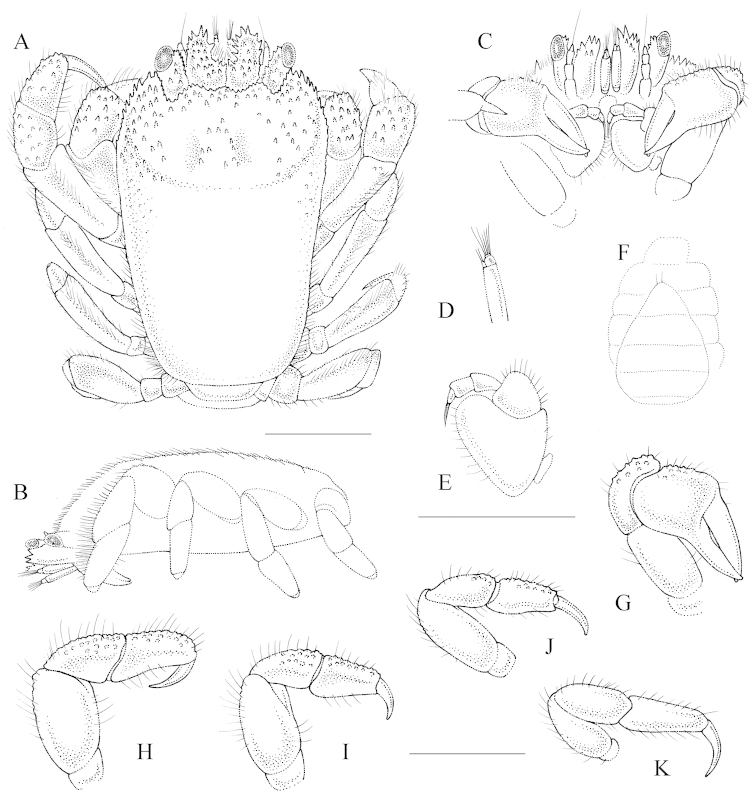
Male allotype (3.6 × 2.5) of *Lithoscaptus semperi* sp. n. (RMNH.Crus.D.56962) **A** habitus, dorsal view **B** carapace, lateral view **C** anterior margin of carapace, ventral view **D** close-up of antennule **E** MXP3 **F** thoracic sternites **G** right P1 (cheliped) **H** right P2 **I** right P3 **J** right P4 **K** right P5. Scale bar 1 mm; A–C, D–E, F–K share scale bars.

Carapace (Fig. 2A) subrectangular to trapezoid, CL 1.5 times longer than CW, widest near anterior half, convex in lateral view, deflected anteriorly, with broad W-shaped depression (Fig. 2A, B). Anterior half of carapace and carapace margins with small spines, posterior half of carapace smooth.

Ocular peduncles with small spines on distal margin, cornea elliptical, longer than broad; antennal article extending beyond eyestalk, with spines along margins (Fig. 2C). Antennule slender compared to holotype, distal segment of antennules with protruding article (Fig. 2D).

MXP3 (Fig. 2E) exopod rectangular; ischium smooth, triangular, few scattered simple setae on distal and lateral margins, merus with distolateral projection, simple setae; propodus, dactylus of similar length, latter with bundle of short setae.

P1 (chelipeds, Fig. 2G) stout; merus length twice height, smooth; carpus with rounded and conical tubercles, simple setae on dorsal surface; propodus stout, with conical tubercles, simple setae on dorsal surface; fingers slender, mesial surfaces of dactyl slightly gaping, tips of fingers crossing.

P2 (Fig. 2H) ischium without setae; merus relatively stout, smooth, length twice height, simple short setae on lateral and dorsal surface; carpus, propodus of similar length; carpus with few rounded tubercles and setae on dorsal surface; propodus smooth except for rounded tubercles on dorsal surface, few setae on lateral, dorsal surface, dactylus smooth, sharp, curved ventrally.

P3 and P4 (Fig. 2I, J) similar to P2, somewhat smaller; ischium without setae; merus smooth, simple short setae on lateral and dorsal surface; carpus, propodus of same length, few rounded tubercles and setae on dorsal surface; dactylus smooth, sharp, curved ventrally.

P5 (Fig. 2K) ischium with few setae; merus, carpus, propodus smooth, with simple short setae on dorsal and lateral surface; dactylus smooth, sharp, curved.

P3, P4 decreasing in size from P2.

Abdomen teardrop-shaped, widest at 4^th^ somite; telson slightly pointed with few simple setae (Fig. 2F).

Gonopod 1 almost straight, tapering, apex sharply pointed. Distal margin with 2-3 non-plumose short simple setae, medial margin without setae (examined in RMNH.Crus.D.56964).

##### Colour.

**Figure 3.**
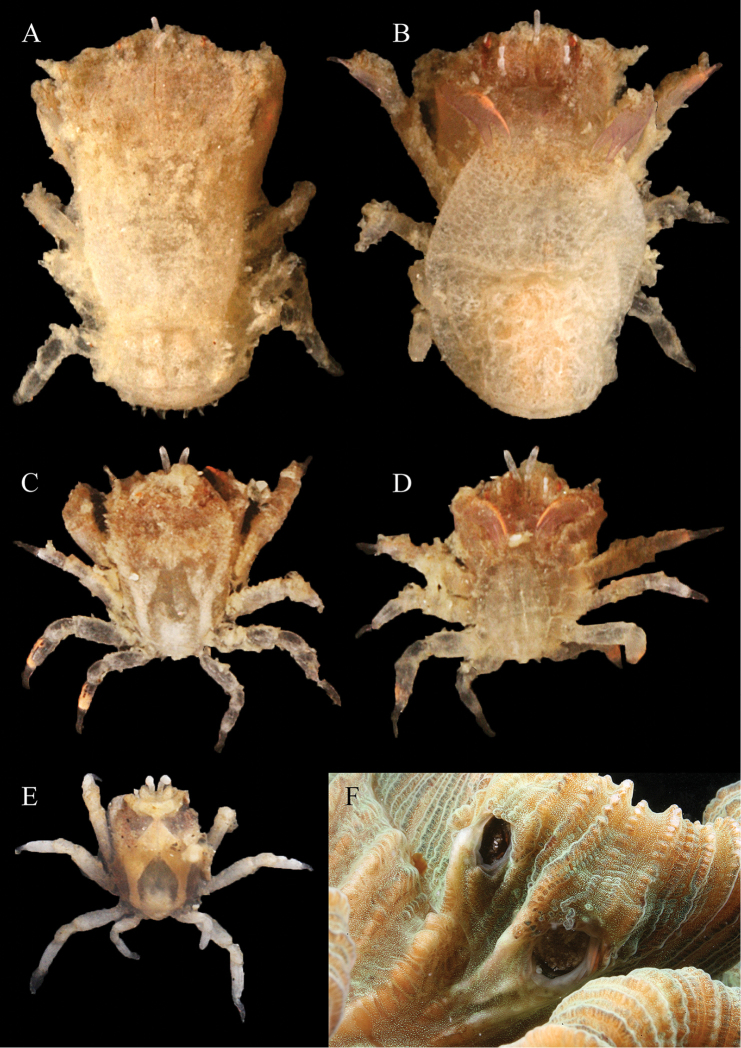
Colour in life of *Lithoscaptus semperi* sp. n. **A–B** non-ovigerous female (4.5 × 3.2; RMNH.Crus.D.54258) dorsal view and ventral view **C–D** male (2.5 × 1.9; RMNH.Crus.D.54258) dorsal view and ventral view **E** juvenile male (2.0 × 1.6; RMNH.Crus.D.56959) dorsal view **F** in-situ photograph of dwellings (left male, right female) of *Lithoscaptus semperi* sp. n. in *Trachyphyllia geoffroyi* on Lubani Rock reef, Kudat (Malaysia). Photos by BT Reijnen/SET van der Meij.

Female (Fig. 3A–B): Overall off-white. Pereiopods opaque, carpus, dactylus P1and P2 translucent violet, sometimes with a pale orange line. Eyes with wide longitudinal brownish-red lines. Male (Fig. 3C–D): Carapace opaque with an off-white distinctive pattern over the whole carapace surface. Pereiopods opaque, P1 carpus, dactylus translucent violet, sometimes with a pale orange line. Eyes brown-red. In juvenile males (Fig. 3E), the carapace pattern is pale orange, pereiopods off-white.

##### Placement in genus.

The placement of *Lithoscaptus semperi* sp. n. in the genus *Lithoscaptus* is somewhat tentative. The first (partial) molecular reconstruction of relationships within the Cryptochiridae shows that the genus *Lithoscaptus* is paraphyletic (van der Meij and Reijnen 2014). However, following the diagnosis of *Lithoscaptus* by Kropp (1990), the new species best fits the genus, except for the absence of a proximal tooth on the cutting edge of P1 dactylus and the presence of a distomesial projection of P2 merus in females. Kropp (1994) noted that his new species, *Lithoscaptus prionotus*, had the pterygostomial region not fused to the carapace, unlike other species in the genus. It is likely that the characters defining the genus need to be redefined, or that certain species need to be moved to a new genus.

##### Comparisons.

Eight species of *Lithoscaptus* are currently recognised (Ng et al. 2008: 212, Davie 2015). *Lithoscaptus semperi* sp. n. can be distinguished from *Lithoscaptus nami* (Fize & Serène, 1957), *Lithoscaptus tri* (Fize & Serène, 1956) and *Lithoscaptus pardalotus* Kropp, 1995 by not having the internal orbital angle extending beyond the external orbital angle. The new species can be separated from *Lithoscaptus grandis* (Takeda & Tamura, 1983), *Lithoscaptus paradoxus* A. Milne-Edwards, 1862 and *Lithoscaptus prionotus* Kropp, 1994 by the smooth appearance of surface of the carapace, despite the small tubercles on the anterior half of the carapace, and the lack of noticeable spines other than the small spines on the frontal carapace margin. *Lithoscaptus pacificus* (Edmonson, 1933) and *Lithoscaptus helleri* (Fize & Serène, 1957) lack the stout merus with prominent distomesial projection of P2 (female specimens). The off-white carapace colour and translucent violet colour on P1 and P2 in females, and the distinctive carapace pattern in males differs from patterns found on other *Lithoscaptus* species.

##### Distribution.

The known distribution of *Lithoscaptus semperi* sp. n. includes northern Borneo and North Sulawesi. Specimens were collected at water depths between 9 and approximately 30 meters. Its host *Trachyphyllia geoffroyi* was described from the Gulf of Suez (Egypt), but this species has a wide distribution that includes the Red Sea, East Africa, Seychelles, Maldives, Nicobar Isls., ‘East Indies’, China Sea, Philippines, Japan, Australia and New Caledonia (Scheer and Pillai 1983). Based on the widespread distribution of *Trachyphyllia geoffroyi*, a wider distribution range than the two presently recorded locations is expected for *Lithoscaptus semperi* sp. n.

##### Coral host.

*Lithoscaptus semperi* sp. n. is so far strictly associated with *Trachyphyllia geoffroyi* (Fig. 3F). It is the first record of associated fauna for this coral host. Colonies of *Trachyphyllia geoffroyi* are free-living, have flabello-meandroid colony shapes and fleshy polyps. Cryptochirids have previously been recorded to inhabit free-living corals; crabs of the genus *Fungicola* are associated with free-living - and attached - mushroom corals (Fungiidae), whereas *Troglocarcinus corallicola* is associated with a wide range of Atlantic corals, including the free-living coral *Manicina areolata* (Mussidae) (Fize and Serène 1957, van der Meij 2014, 2015).

##### Remarks.

Fize and Serène (1957: p. 163) report on *Cryptochirus coralliodytes* from *Trachyphyllia* based on a record of Semper (1881: p. 221) who writes: “*I found them* [Cryptochirus coralliodytes] *in the Philippine Archipelago in cavities in* Goniastraea Bournoni [= Goniastrea retiformis (de Lamarck, 1816)], *in an undetermined true* Astræa, *which was unfortunately lost, also in an undescribed* Trachyphyllia; *finally I received a new form through A. Agassiz from the West Indian seas, which may perhaps form a distinct genus, though it is very nearly allied to the first. It also lives in a* Trachyphyllia.” The coral genus *Trachyphyllia* is described from the Red Sea and has a widespread Indo-Pacific distribution; however, it does not occur in the Atlantic Ocean. The most similar Atlantic species would be *Manicina areolata* (Linnaeus, 1758). Furthermore, on p. 453 (note 103 belonging to p. 221) Semper writes: “*This crab, living in* Trachyphyllia, *a West Indian coral, is extremely like* Cryptochirus, *and perhaps belongs to the same genus; this can only be determined by future and more exact examination. But the ‘cave dwelling’ of this West Indian crab is perfectly unlike that of the Eastern species, which is found from the Red Sea as far as the Pacific Ocean; it is not cylindrical, but has one side quite flat, so that its transverse section is almost exactly a half-circle; the underside of the crab rests against the flat side of the cavity.*” The gall crab *Troglocarcinus corallicola* Verrill, 1908 has been recorded from a wide range of hosts, including *Manicina areolata* (Kropp and Manning 1987, van der Meij 2014). As mentioned by Semper (1881), the dwelling of *Troglocarcinus corallicola* in *Manicina areolata* is shaped like a half-circle (see e.g. Van der Meij 2014: Fig. 1B); therefore, it seems plausible that Semper was referring to the coral *Manicina areolata* when he discussed a West Indian *Trachyphyllia*. Alternatively, Semper could have been referring to the Atlantic genus *Colpophyllia* because Milne Edwards and Haime (1849), who established *Trachyphyllia*, compared their new genus with *Colpophyllia* (see Huang et al. [2014] for a discussion on the genus *Trachyphyllia*). Like *Manicina areolata*, *Colpophyllia natans* (Houttuyn, 1772) also hosts *Troglocarcinus corallicola* (see van der Meij 2014). It remains unclear whether Semper found gall crabs in Indo-Pacific corals currently recognized as *Trachyphyllia geoffroyi*. Semper is not known to have formally described any gall crab species (Ng et al. 2008).

##### Etymology.

Named after the German naturalist Carl Gottfried Semper (1832–1893), who was the first to mention gall crabs occurring in *Trachyphyllia*.

## Supplementary Material

XML Treatment for Lithoscaptus semperi
